# Exploring the lived experiences of women living with HIV in rural Zimbabwe: A qualitative study

**DOI:** 10.1097/MD.0000000000039485

**Published:** 2024-09-06

**Authors:** Limkile Mpofu, Makombo Ganga-Limando

**Affiliations:** a Department of Health Studies, Faculty of Human Sciences, University of South Africa, Pretoria, South Africa.

**Keywords:** HIV/AIDS, interpretative phenomenological analysis (IPA), qualitative, rural Zimbabwe, stigma and discrimination

## Abstract

The study of human immunodeficiency virus (HIV)-related stigma and discrimination has been burgeoning with important implications for public health and society, as it negatively impacts people living with HIV. However, data on the experiences of rural women living with HIV/Acquired Immune Deficiency Syndrome (AIDS) in Zimbabwe are lacking. Women represent 50% of the global pandemic, while deaths from AIDS-related illnesses have exceeded 35 million. This study aimed to explore the experiences of rural women living with HIV/AIDS in Zimbabwe. Forty rural women living with HIV were selected from 6 villages (one village per district) of Matabeleland South Province in Zimbabwe. A qualitative descriptive research design using in-depth individual interviews from 22 purposefully selected rural women living with HIV and 3 focus groups, was used to collect the study data. The transcripts of the interviews were analyzed using interpretative phenomenological analysis. Three interconnected themes were identified: social prejudice, social discrimination, and psychosocial dysfunction. A key finding in the themes was that women living with HIV in rural Zimbabwe were psychosocially dysfunctional because of social prejudice and discrimination perpetrated against them by significant others in their communities. The findings provide a valuable understanding of women’s experiences of living with HIV and AIDS in Africa’s low-income countries. These results can be used by researchers, clinicians, mental health providers, and policymakers to address the unique needs of rural women living with HIV/AIDS.

## 1. Introduction

Stigmatization associated with human immunodeficiency virus (HIV) and Acquired Immune Deficiency Syndrome (AIDS) continues to be a global problem, even after 40 years of the pandemic.^[1]^ The stigma, discrimination, and unfair treatment of people living with HIV have a significant negative impact in many communities, especially rural settings.^[2]^ Social exclusion and stigmatization contribute to mental health issues and resistance to adherence, resulting in the spread of HIV among people and the progression of HIV. People living with HIV/AIDS (PLHIV) don’t seek early treatment due to stigma and discrimination, which could lead to poor adherence to treatment, low quality of life, or increased risk of violence.^[2,3]^ Most Zimbabwean residents reside in rural areas (86%), still high-risk populations.^[4]^ Rural women living with HIV and AIDS face enormous challenges, including HIV-related stigma and mental issues.^[5]^ Women living in rural areas of Zimbabwe are affected by HIV more than those living in urban areas are—for example, the prevalence rate of HIV in the rural province of Matabeleland South, where the study took place, was 21.6% compared to 12.2% in Harare.^[6]^ The male-dominated nature of society and employment opportunities are often attributed to this high prevalence rate. Authors Pretorius and colleagues argue that women’s sexual and reproductive health rights are usually not respected in a male-dominated society.^[7]^ This situation deprives women of deciding on sexual issues affecting their health. A report by UNAIDS showed that 32.0% of men believe a man has a right to have multiple partners.^[8]^ For instance, more than a quarter of women with a married or stable partner have experienced physical or sexual violence from their partner.^[8]^ Again, a lack of employment opportunities in rural areas compels most men to leave their wives, families, and rural communities behind to look for employment in urban areas, where they end up staying with another woman.^[7]^

In third-world countries, land is essential and considered to be the identity of the people and the very life of the rural populations^[9]^ Chigbu and colleagues posit the “situation as a reality in many sub-Saharan countries, especially Zimbabwe, where customary land tenure systems actively govern access to land”^[10]^ As the men would have left for urban areas, the rural women became the driving force in agriculture. They till the land; however, despite these women producing most of the world’s food, they have limited control over ownership or access to land.^[10]^

Culturally, society expects women to be faithful to their husbands, both physically and socially. Simultaneously, their men start other families in towns.^[11]^ Again, women who stay home take up additional roles without the support a physically present husband typically provides.^[7]^ This role adjustment may influence women’s living experiences and responses to social demands.^[7]^

Women living with HIV in rural areas adjust to these new social role expectations. These women are also likely to live well with stigma and discrimination related to their HIV-positive status. Researchers argue that stigma and discrimination are socially constructed and expressed through social interaction.^[12,13]^ That is, stigma is constructed through social relationships, which means that the rules guide behavior by defining it as acceptable, customary, “normal,” or expected at particular points in time and place. As such, facilitating these social, interpersonal, and structural injustices should be a social priority with the end goal of their elimination.^[12]^ Stigma and discrimination take place within a context of differential power between the stigmatizing and stigmatized group; that is, involving stereotypes, the endorsement of those stereotypes as accurate (prejudice), and a desire to avoid or exclude the stigmatized persons (discrimination).^[14]^ As a socially constructed concept, social values, norms, and beliefs influence how people respond to stigmatized behavior.^[15]^ Thus, women living with HIV in Zimbabwe’s rural areas need to live in communities where people share the same social values, norms, and beliefs.

From the preceding context, it becomes essential for the researchers to renew their attention and focus on stigma and discrimination alleviation topics and additional research to understand how these women live in such an environment to design intervention strategies. Besides, as women in rural areas are the higher driving force in agriculture^[10]^ and have a higher HIV prevalence than men,^[6]^ their neglect may mean a significant decrease in agriculture. Hence, this research will be a value addition, aiming to explore the lived experiences of rural women living with HIV in Zimbabwe. UNAIDS’ Global AIDS Monitoring of 2018 revealed some indicators for monitoring the 2016 United Nations political declaration on ending AIDS.^[16]^ Within these global indicators were commitments specifying eliminating discrimination against women, girls, and PLHIV, which makes this research highly pertinent. This study provides knowledge that can be used to raise public awareness of the psychological needs of women. It offers practical information to support researchers and policymakers in developing family-focused interventions and education programs for rural women in Zimbabwe. These would be education programs that provide information about living with PLHIV and how to avoid HIV-related stigma.

## 2. Methods

### 2.1. Study design

This study employed an exploratory, descriptive design. An Interpretative Phenomenological Analysis (IPA)^[17]^ was adopted to explore and describe women’s experiences living with HIV and AIDS in rural Zimbabwe. The women’s lived experiences helped construct the meaning as they encountered their personal experiences^[18]^ with HIV-related stigma. The phenomenological interpretative design and grand tour questions allowed the participants to freely account for their personal experiences from their perspectives rather than an objective truth.^[18]^ Relevant behavioral outcomes, referents, cultural factors, facilitators, and barriers for each particular behavior target population under investigation were identified.

### 2.2. Participants and setting

A total of 40 women living with HIV (22 in-depth interviews and 3 focus groups with 6 people per group) from 6 villages of Matabeleland South Province were recruited using purposive sampling.^[19]^ Matabeleland South Province was selected because of the high prevalence rate of HIV (21.6%) and the social, cultural, and language homogeneity of the population.^[4]^ The geographical situation was also crucial for this study as most males in these bordering areas migrate to South Africa for employment. Participants were included if they were 18 years and above, diagnosed HIV positive for at least 1 year from the date of the data collection, living in the villages for at least 1 year since the time of being diagnosed HIV positive plus on antiretroviral therapy (ART) for at least 6 months, and being able and willing to give a good account of living with HIV/AIDS. The final sample contained women with different clinical stages of HIV, ages, marital statuses, villages in the province, and levels of education and employment status to achieve the maximum difference in sampling as per the community workers’ register books with details of all women taking ART. The in-depth interviews were conducted in a natural setting (participants’ homes/households), which enabled the participants to become familiar with the researcher and open up during the meeting.^[17]^ However, participants of focus groups opted for venues at the nearest clinics. The participants used these clinics to collect their antiretroviral drugs and attend support group meetings for people living with HIV. The researchers secured the focus group venues with the support of the community health workers.

### 2.3. Data collection

Data were collected through semi-structured interviews and focus groups between July and August 2019. The interviews were conducted in English (as per the majority of participants’ choice but with an option to express themselves in the local language [Ndebele] when needed) at their own homes and clinics. Three grand tour questions guided the discussions for both individual interviews and focus group discussions. These questions examined the experiences of a woman living with HIV in rural Zimbabwe’s social context and how they respond to HIV-related stigma and discrimination; such questions are exemplified here**—**“Tell me about your life experiences as a woman living with HIV in this village.” And**—**“What does it mean to you as a woman living with HIV in this village?.” Probing questions were also raised based on interviewees’ responses to enhance the richness of the data. “Please explain more. What does that mean to you as a woman living in this community?” Such questions also probed their experience with social prejudice and discrimination and how it affected their psychosocial functioning and well-being. By detailing their experiences and what it meant to them to live with HIV as a woman, the participants also pointed to their coping strategies or resilience factors that they used to deal with stigma and discrimination. Depending on their willingness and tolerance, in-depth interviews varied between 40 and 55 minutes, while focus groups lasted an average of 90 minutes. The researchers explained the research objective and ethical considerations and obtained the participants’ informed consent and permission to record the interviews. The interviews were checked for quality and transcribed verbatim. The key findings were discussed within 24 hours.

### 2.4. Data analysis

To “give voice” to the participants’ life viewpoints, the researchers examined rural women’s lived experiences using IPA to analyze the transcripts.^[20]^ Thus, the researchers conducted an in-depth inductive qualitative analysis. The researchers used the IPA method’s step-by-step transcript analysis to code and analyze the transcripts.^[21]^ The process entailed organizing, coding, integrating, and interpreting data.^[22]^

During the text review, the authors focused more on the subjective point of view of the participants than on factual data. To familiarize themselves with the data, the authors read and reread each participant’s transcript separately. This process was repeated several times for all transcripts, enabling the author to obtain a holistic overview of the participant’s thoughts and feelings through their stories. The authors recorded their observations and comments at this phase, as in Smith and Osborn^[23]^ interpretative phenomenological analysis.

In the next phase, the author coded each interview transcript line-by-line. Important descriptive, linguistic, and conceptual themes for each transcript emerged by focusing on connections across the themes for every narrative and then across the descriptions. Thus, data collection was fulfilled simultaneously with data analysis until the interviews produced no new information, thereby reaching theoretical saturation.^[23]^

#### 2.4.1. Rigor

The authors reviewed and developed the analysis to improve the validity and reliability of the findings. The supervisor’s insights into the analysis were incorporated as an independent reviewer to establish the results’ credibility and accuracy. After iterative review and discussion phases, the analysis of each interview resulted in overall agreement regarding the final examination and written report. The findings are described with relevant narratives from the rural women living with HIV/AIDS, bearing in mind that these women had all opted for English with the option to express themselves in Ndebele when needed. Thus, enabling triangulation and enriching the analysis as a quality control procedure. Again, as researchers, we became aware of our contribution to constructing meanings and lived experiences throughout the research process through reflexivity. Thus, the researchers had their own reflexivity notes/insights revealing how they explored how their involvement in this research influenced or informed this study. So, reflexivity was part of the authors’ commitment to ensure trustworthiness.

### 2.5. Ethical considerations

The study was approved by the Research and Ethics Committee of the University of South Africa (UNISA) (HSHDC/847/2018) and the Medical Research Council of Zimbabwe (MRCZ) with approval reference (MRCZ/A/2398) before the researchers commenced data collection. All participants provided written informed consent before data collection, while data collected were anonymized and kept strictly confidential to be used for research purposes only.

## 3. Results

Forty rural women living with HIV (WLHIV) (mean age 40.93 years, SD 8.57) were recruited and interviewed. Twenty-two were face-to-face interviews, while 18 went for focus group discussions. There were 6 participants per group to make 3 focus groups. Among the 40, 21 women had gone up to the primary level, 12 high school, and 7 tertiary education. Regarding their relationship status, single, divorced, or widowed counted up to 26, while married women were 14. Under the unemployed or retired category, we had a high number—27 women. Thirteen of the women were at least employed either part-time or full-time. Their time since they were diagnosed HIV positive was similar to their time since they enrolled for ART. Characteristics of the 40 study participants are summarized in Table 1.

**Table 1:** Characteristics of the study participants (N = 40).

Characteristics	N	%
Age, years		
20 to 27	2	(5)
28 to 35	8	(20)
36 to 43	16	(40)
44 to 51	9	(22.5)
52 to 59	5	(12.5)
Marital status		
Single/divorced/widowed	26	(65)
Married	14	(35)
Education		
Primary or below	21	(52.5)
Secondary	12	(30)
Tertiary	7	(17.5)
Employment		
Unemployed or retired	27	(67.5)
Employed	13	(32.5)
Time since HIV positive and ART initiation		
+6 years	12	(30)
4–6 years	21	(52.5)
0–3 years	7	(17.5)

Data analysis revealed 3 major themes of being HIV-positive women in rural Zimbabwe: Social prejudice, social discrimination, and psychosocial dysfunction. These concepts were related to personal or social factors. Thus, from these accumulated lived experiences, *it was deduced that women living with HIV in rural Zimbabwe are psychosocially dysfunctional because of the social prejudice and social discrimination perpetrated against them by significant others in their communities.*

The 3 themes that appeared to portray being HIV-positive women in rural Zimbabwe are as in Table 2.

**Table 2:** Summary of themes and related sub-themes.

Themes	Subthemes
Social prejudice	1. Blame and shaming
	2. Social avoidance
	3. Social rejection
	4. Undignified treatment
	5. Gender stereotype
Social discrimination	1. Loss of independent decision-making power
	2. Unfair labor practice
	3. Gender-based discrimination
Psychosocial dysfunction	1. Blaming oneself
	2. Cognitive avoidance
	3. Hopelessness
	4. Worthless
	5. Social isolation or withdrawal

### 3.1. Theme 1: Social prejudice

Social prejudice as a new social reality for women living with HIV in rural Zimbabwe refers to any dehumanizing behavior displayed by significant others toward women living with HIV because of their HIV-positive status. Women living with HIV experienced these behaviors contrary to their communities’ accepted prevailing socio-cultural norms.

Five subthemes were derived from the participants’ descriptions of behaviors they experienced as dehumanizing. It emerged that the interaction between women living with HIV and their significant others in the community was characterized by (1) blame and shaming, (2) social avoidance, (3) social rejection, (4) undignified treatment, and (5) gender stereotypes. The participants identified significant others as perpetrators of these dehumanizing behaviors, including partners, close family members, in-laws, friends, and the community.

From the above lived social prejudice experiences, *it was deduced that women living with HIV in rural Zimbabwe are socially victimized and treated in a degrading manner for being HIV positive and women.*

Below are direct quotes/ narratives from each subtheme under social prejudice:

A 42-year-old woman was treated by her mother-in-law as defiant and uncaring for bringing AIDS to her matrimonial home. She felt so bad that her mother-in-law told her children that she had killed her husband because of her insensitive behavior toward her family.

*My biggest concern and pain is how my mother-in-law treated me after my husband’s death due to HIV and AIDS. She wouldn’t do that to her son, but she blames me for being responsible for the death of her son. She even told my children I killed my husband because of my uncaring and defiant behavior.* (in-depth individual interview [IDI] 04)

The social avoidance behavior displayed by close family members was based on the belief that a person living with HIV can quickly transfer the disease to family members through physical contact and sharing things. In one of the focus groups, some of the participants narrated:

*You see, even our family members see us as people with the potential to infect everybody in the household.* (FGD 01)

Social rejection was often expressed through the discontinuation of relationships that the participants enjoyed before being tested HIV positive. Significant others initiated this discontinuation without giving any reasons besides their HIV-positive status. Partners, in-laws, friends, and church gatherings mainly perpetrated social rejection.

*My husband divorced me after I tested HIV-positive. Lucky for him, he tested HIV negative. I told him he must look after himself and live without me. I tried everything to keep him, but I failed. He clearly said to me that he could not continue his life with an HIV-positive woman.* (IDI 20)

*My in-laws told me to move and set up our homestead after my husband had died.* (IDI 16)

The experiences of the participants in this study were described with a lot of emotions and disbelief. It was best captured with the following narrative from a 40-year-old married woman:

*Things that bothered us the most were the lack of consideration of our humanness. They talk to you as if you are not a human being. We are treated like lepers or polluted sources that need to be dumped far away. We are treated as outcasts and do not have the right to belong and be respected.* (IDI 04)

### 3.2. Theme 2: Social discrimination

Social discrimination as a theme referred to any social behavior displayed by significant others toward women living with HIV, which they experienced as violating their individual and social rights because of their HIV-positive status. Women suffered social discrimination at the hands of their partners, close family members, in-laws, friends, and the community.

Three sub-themes emerged from the analysis of the participants’ descriptions. These were classified as (1) loss of independent decision-making power, (2) unfair labor practices, and (3) gender-based discrimination.

From the above lived social discrimination experiences, *it was deduced that women living with HIV in rural Zimbabwe are denied their social rights because of their HIV-positive status and gender.*

The following statements bear evidence:

From the descriptions of their experiences, women were deprived of their rights to make independent decisions on their education, sexual and reproductive health, family, and social matters.

*It is so sad how people can decide on an important matter that concerns your future without your involvement. I was in my first year of university when I was tested HIV positive. After sharing the news with my legal guardian, who was paying for my studies, he told me to return home. He stated that he would not waste his money paying for the education of someone who is HIV positive. So, when I finally came home, he forced me to marry an older man. He said marrying an older rich man would boost the family’s financial status.* (IDI 03)

The lack of decision-making roles affected most women without a male child. It meant that the WLHIV had no land rights, which affected their financial freedom as they would have to depend on the male surviving relatives. This is what transpired from one of the FGDs.

*You know, when your husband dies of HIV and AIDS-related disease, you, as a surviving spouse, have nothing on your name. I don’t have a male child, and our community disregards the number of years we have lived with our husbands and go on to strip us of our land. All decisions are taken for us. We are not given an opportunity to express our opinions in this community.* (FGD 02)

Unmarried women living with HIV felt discriminated against because of being unmarried and HIV positive. A 41-year-old woman narrated:

*There is a lack of recognition as an equal community citizen. Mostly, you have to be married to earn respect.* (IDI 15)

### 3.3. Theme 3: Psychosocial dysfunction

Psychosocial dysfunction as a theme refers to a set of harmful cognitive, emotional, and social mechanisms used by women living with HIV in response to the social prejudice and discrimination perpetrated against them by significant others in their communities.

Women living with HIV were confused about effectively dealing with the social prejudice and discrimination perpetrated against them by significant others. The extract below from a 49-year-old married woman exampled the state of confusion experienced by most women living with HIV in rural Zimbabwe:

*Knowing my status affected me psychologically. The thought of the community’s boundaries regarding how I should love my children saddened me. Because they dictate and limit the interaction between my children and me, I needed to be careful about how I mingle with them. People’s eyes are always on me in the name of protecting the children. I could feel the scorn in their voice and face. People are so bitter. I don’t know what I have done wrong.* (IDI 10)

Five sub-themes emerged from the qualitative analysis of the participants’ descriptions. These included: (1) blaming oneself, (2) cognitive avoidance, (3) hopelessness, (4) worthless, and (5) social isolation/withdrawal.

From the above-lived experiences, *it was deduced that women living with HIV in rural Zimbabwe developed a set of cognitive, emotional, and social dysfunction due to social prejudice and discrimination perpetrated against them by significant others in the communities where they live.*

Below are examples from each sub-theme under psychosocial dysfunction:

A 33-year-old unmarried woman expressed inward anger for being HIV positive and could not have children because of gender-based discrimination.

*I am angry at myself. I feel sad and disappointed with myself. I am unmarried and will never have children because of what people think of an HIV-positive woman in this community.* (IDI 07)

Avoidance to talk or discuss anything related to HIV and AIDS was best captured from an extract from a 50-year-old married woman. Her husband was also living with HIV. Despite being HIV positive, they deliberately decided not to talk or discuss HIV matters in their matrimonial home.

*My husband and I do not talk or discuss HIV or AIDS in our house. My husband believes that only prostitutes speak about HIV and AIDS. So, we do not talk about it in this home.* (IDI 12)

A 30-year-old unmarried woman narrated how she avoids taking on specific responsibilities or engaging in friendships because of social prejudice against women living with HIV in their community.

*I do not even consider taking any responsibility or making friends because of how people treat us in this community. I avoid going through the humiliation of being HIV positive. It is horrible because they treat you like a sick person who is mentally unstable.* (IDI 17)

Despite access to antiretroviral drugs, women living with HIV did not have hope for the future. A 36-year-old married woman expressed her state of hopelessness as follows:

*I had a completely new outlook on life after I tested HIV positive. I do not look that far into the future anymore. I don’t look that far ahead. I do not think about getting old. Even though there are antiretroviral drugs, there’s a possibility that I won’t get to see my grandchildren.* (IDI 02)

Those feelings were associated with suicidal ideation, as narrated by a 44-year-old woman in FGD 03.

*Some of us are just living because we are still breathing. As for me, I am as good as dead. I have been sick for some years. It is better to die. My husband has since died, but I am still living. I wish it were me who had died first.* (FGD 03)

It emerged that women living with HIV found it difficult to interact with significant others because of the social prejudice against them by members of their communities. They turned to social isolation or withdrawal to avoid social discrimination by significant others in their communities.

A 41-year-old married woman shared her experience with sadness:

*I decided not to interact with people in my community because of their negative behavior toward people living with HIV. It is painful, indeed. I contemplated leaving this village several times to escape the shame and judgment. I couldn’t cope with the torture of being reminded of my HIV status. I always stayed behind closed doors.* (IDI 15)

The following framework summarizes the narratives above.

## 4. Discussion

**Figure 1.**
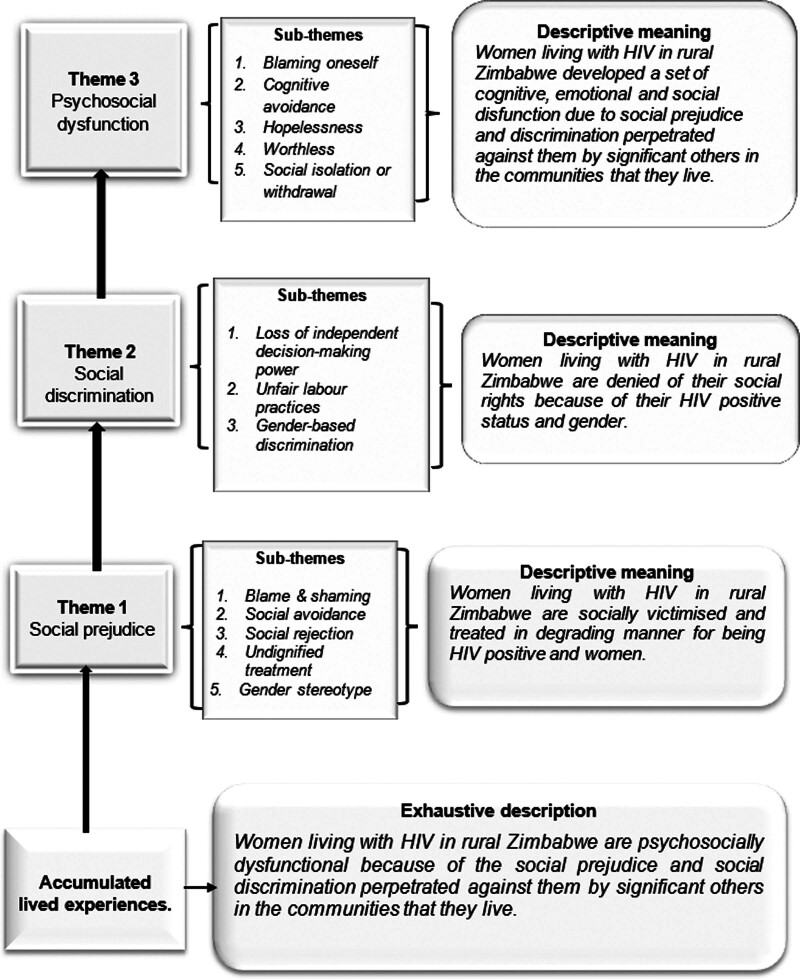
Framework of the lived experiences of women living with HIV—author’s own construction.The base boxes in this figure represent the accumulated lived experiences with the exhaustive description that reflects the meaning of being a woman living with HIV in this community. The level that follows the base boxes is theme 1, which is made up of the sub-themes in the box next to it. Then, the descriptive meaning is deduced from the women’s excerpts. Theme 1 is followed by theme 2 boxes, sub-themes, and the deduced descriptive meaning. Theme 3 then follows with its sub-themes and descriptive meaning, as highlighted by the participants. The black arrows indicate the main themes that make up the accumulated lived experiences.

The present study explored the Zimbabwean rural women’s first-hand experiences of living with HIV. The findings showed that women living with HIV in rural Zimbabwe are denied their social rights because of their HIV-positive status and gender. As in the framework in Figure 1, the first theme to emerge from this study involved women being socially victimized and treated in a degrading manner for being HIV positive and women. Such victimization supports previous studies that portray the role of significant others (partners, close family members, in-laws, friends, and the community) as the drivers of social stigma and discrimination.^[7]^ This is also in line with the social nature of stigma and discrimination. As socially constructed concepts, stigma and discrimination are perpetrated by significant others in the social environment of the victims^[23]^ and shaped by the cultural beliefs and norms governing society.^[24]^ This is similar to this study’s findings, where women living with HIV were judged and labeled according to the cultural values and norms of the communities.

The second theme identified in the current study captured the denial of women’s social rights because of their HIV-positive status and gender. Thus, gender stereotypes and gender-based discrimination pointed to the prevailing patriarchal nature of society.^[25]^ In the current study, social prejudice and discrimination perpetrated against women living with HIV were not the same as that of men living with HIV. Scholars accede to this practice of social prejudice and discrimination against WLHIV and allude that most women were abandoned by their families and society because of the HIV-related stigma and discrimination.^[2,26]^

The final theme described women living with HIV in rural Zimbabwe as self-inflated with stigma and discrimination, resulting in psychosocial dysfunction expressed through blaming oneself, cognitive avoidance, hopelessness, worthless, and social isolation or withdrawal. This is consistent with previous studies.^[27,28]^ Again, previous qualitative studies have suggested that self-stigma is related to various negative emotions, such as stress, guilt, and shame,^[25,29]^ the risk factors for depression.^[30]^ Therefore, a deeper insight into the psychological and social determinants of health of rural WLHIV is needed. The study revealed that women living with HIV in rural Zimbabwe are psychosocially dysfunctional because of the social prejudice and discrimination perpetrated against them by significant others in their communities. Community support systems are crucial for support networks or family-focused interventions within our community. The access to empowerment opportunities for women living with HIV and women in resource-limited environments is associated with economic independence and reduced gender equality across the globe^[31]^ It is important to foreground the debate on women’s financial freedom to increase women’s capacity to make real-life choices through full and equal participation in all spheres of life.^[32]^ The current study presents evidence that supports UNAIDS’ global indicator commitments to eliminate discrimination against women, girls, and people living with HIV/AIDS. Besides, the study is conducted in a rural setting, which provides good ecological/external validity as it enables one to view or take the findings in their natural/cultural context.

Since the present study only focused on rural women living with HIV in Matabeleland South Province, the results might not be fully generalizable and assumed to apply to other rural women within this population across various contexts. Future studies must test a wider geographical region on the diverse rural women’s experiences in Zimbabwe. In our case, however, data were collected using a flexible semi-structured interview schedule, grand tour questions, focus groups, and in-depth analysis, which means that rich and detailed data were obtained regarding the experiences of these rural women.

Given that the rural woman living with HIV in this study reported feelings of self-blame, anger, and guilt for being HIV, it may be necessary for mental health providers to proactively offer psychotherapy education to illustrate behavioral strategies and explain that HIV is not a death sentence to these rural women. Such teachings might reduce rural women living with HIV’s stress and increase mental well-being. From a social perspective, policymakers need to establish preventive education programs to improve public awareness and foster people’s more profound understanding of the characteristics and life-long needs of individuals living with HIV and AIDS. Learning to accept people living with HIV may help the community increase positive societal attitudes toward people living with HIV and reduce the social stigma and discrimination associated with people living with HIV and AIDS, as nonacceptance impedes the inclusion of individuals living with HIV and overall social life.

## 5. Conclusion

This study provided valuable insights into the rural Zimbabwean women’s lived experiences of living with HIV and AIDS. The current study revealed that women living with HIV in rural Zimbabwe are psychosocially dysfunctional because of the social prejudice and social discrimination perpetrated against them by significant others in their communities.

The social HIV-related stigma and discrimination were classified as social prejudice and social discrimination. Significant others perpetrated them within the family, workplace, schools, churches, healthcare settings, and the community social network.

From the qualitative analysis of the women’s lived experiences, it emerged that women living with HIV in rural Zimbabwe were socially victimized and treated degradingly. It denied their social rights because of their HIV-positive status and gender. In response to social prejudice and discrimination, women living with HIV in rural Zimbabwe developed a set of cognitive, emotional, and social dysfunction. The researchers used the above findings to create a framework of women’s lived experiences living with HIV in rural Zimbabwe (see Fig. 1). As illustrated in Figure 1, this framework included the themes, sub-themes, and the specific and descriptive meaning of the emerged experiences.

## Acknowledgments

The authors would like to thank all the rural women living with HIV in Matabeleland South Province who have participated in the study, the clinics for the venue of the focus groups, the health village workers who facilitated access to the key informants, Jaylee Group (Pty) LTD for sponsoring the PhD programme from which study was extracted, the University of South Africa (UNISA), and the Research Committee for approving the PhD research entitled “Model for mitigating stigma and discrimination against women living with HIV in rural Zimbabwe” as supervised by Professor Ganga-Limando.

## Author contributions

**Conceptualization:** Limkile Mpofu, Makombo Ganga-Limando.

**Data curation:** Limkile Mpofu, Makombo Ganga-Limando.

**Formal analysis:** Limkile Mpofu, Makombo Ganga-Limando.

**Funding acquisition:** Limkile Mpofu, Makombo Ganga-Limando.

**Investigation:** Limkile Mpofu.

**Methodology:** Limkile Mpofu, Makombo Ganga-Limando.

**Project administration:** Limkile Mpofu.

**Resources:** Limkile Mpofu.

**Software:** Limkile Mpofu.

**Supervision:** Makombo Ganga-Limando.

**Validation:** Limkile Mpofu, Makombo Ganga-Limando.

**Visualization:** Limkile Mpofu.

**Writing – original draft:** Limkile Mpofu, Makombo Ganga-Limando.

**Writing – review & editing:** Limkile Mpofu, Makombo Ganga-Limando.
